# Lactate Is Answerable for Brain Function and Treating Brain Diseases: Energy Substrates and Signal Molecule

**DOI:** 10.3389/fnut.2022.800901

**Published:** 2022-04-28

**Authors:** Ming Cai, Hongbiao Wang, Haihan Song, Ruoyu Yang, Liyan Wang, Xiangli Xue, Wanju Sun, Jingyun Hu

**Affiliations:** ^1^Department of Rehabilitation Medicine, Shanghai University of Medicine and Health Sciences Affiliated Zhoupu Hospital, Shanghai, China; ^2^Bio-X Institutes, Shanghai Jiao Tong University, Shanghai, China; ^3^Department of Physical Education, Shanghai University of Medicine and Health Sciences, Shanghai, China; ^4^Central Lab, Shanghai Pudong New Area People's Hospital, Shanghai, China; ^5^College of Rehabilitation Sciences, Shanghai University of Medicine and Health Sciences, Shanghai, China; ^6^Key Laboratory of Exercise and Health Sciences of Ministry of Education, Shanghai University of Sport, Shanghai, China

**Keywords:** lactate, brain function, brain diseases, energy substrates, signal molecule

## Abstract

Research to date has provided novel insights into lactate's positive role in multiple brain functions and several brain diseases. Although notable controversies and discrepancies remain, the neurobiological role and the metabolic mechanisms of brain lactate have now been described. A theoretical framework on the relevance between lactate and brain function and brain diseases is presented. This review begins with the source and route of lactate formation in the brain and food; goes on to uncover the regulatory effect of lactate on brain function; and progresses to gathering the application and concentration variation of lactate in several brain diseases (diabetic encephalopathy, Alzheimer's disease, stroke, traumatic brain injury, and epilepsy) treatment. Finally, the dual role of lactate in the brain is discussed. This review highlights the biological effect of lactate, especially L-lactate, in brain function and disease studies and amplifies our understanding of past research.

## Introduction

Brain lactate, as a well-known metabolite, primarily roots in astrocytic glycolysis from blood glucose, glycogen, and blood lactate. Recently, the role of the “good guy” has gradually superseded the traditional concept of metabolic waste in medical literature in neuroscience (1). The most interesting dimension of this is the physiological character of lactate's role in mediating brain function (2). These canonical function involves learning and memory (3), cerebral blood flow (4), neurogenesis (5, 6) and cerebral microangiogenesis (7), energy metabolism (8), neuronal activity (9–11), and neuroprotection (12–15). Therefore, lactate is competent to be a potential therapy for ameliorating the pathological process of some brain diseases associated with impaired brain function. In mammals, lactate exists as two enantiomers. The structure of asymmetrical C2 carbon leads to the two stereoisomers of lactate that are designated as L-lactate and D-lactate (16). L-lactate is the major enantiomer found in the brain and blood whereas D-lactate is normally present in very low concentrations under healthy physiological conditions (17). D-lactate is also considered as the rivalrous inhibitor of L-lactate since it competitively inhibits L-lactate transport (18). In different brain disease patterns, L- and D-lactate is reported to exert a similar or distinct effect on brain function. The involving mechanisms are far more complex than originally thought. For the most part, L-lactate can be utilized as a preferred energy substrate of neurons for meeting the energy demand (19, 20) or act as the novel hormone-like effect called lactormone (21, 22). But current research about D-lactate's role in brain function and brain-related disease is sparse and debatable. D-lactate-mediated mechanisms are also unclear.

In this narrative review, we aim to provide a comprehensive and profound summary of the role of lactate in brain function and related diseases. Consequently, we expound the food source of lactate intake, discuss the lactate enantiomers and their metabolism manner in the brain, compare the influence of L- and D-lactate on brain functions, expound on the effect of L- and D-lactate replenishment on several common brain diseases, and summarize the mechanisms of L-lactate.

## Lactate Enantiomers

Lactate in mammals exists as two enantiomers: the most common form of L-isomers (known as L-lactate) is produced during mammalian glucose metabolism, and a quite small quantity of D-isomers (known as D-lactate) is generated from carbohydrates by bacterial metabolism (23, 24). The L-isomer of lactate is believed to have biological metabolic activity, while the D-isomer is too low in the body to activate the relevant enzymes for catabolism (25).

### The Formation of Lactate in the Brain

#### L-Lactate

In the brain, the main source of L-lactate is astrocytic glycolysis from blood glucose, glycogen, and blood lactate (26, 27). In astrocytes, glucose can be converted directly to L-lactate by glycolysis or be stored in the form of glycogen (28). Glycogen is almost exclusively localized in astrocytes (20). As the neuronal activity intensifies, astrocytic glycogen is mobilized to supply neurons in case of neuronal glucose dissatisfying the energetic demand (29). In consequence, besides maintaining the astrocytes itself energy demand, L-lactate also supports neuronal activity by providing ATP (30, 31). Furthermore, the elevated blood L-lactate can also cross the blood-brain barrier into the brain *via* the monocarboxylate transporter 1 (MCT1) in some conditions (32, 33), such as vigorous exercise (34, 35) and fermentative (36) or fiber-containing foods (37).

#### D-Lactate

D-lactate, as the stereoisomer of L-lactate, is barely found in the brain and does not participate in energy production (23). A tiny amount of methylglyoxal (MG), the metabolic intermediary product, can be produced during glycolysis (38). The glyoxalase system, mostly located in astrocytes, allows bulk MG to convert into endogenic D-lactate or glutathione (GSH) (39).

### The Metabolism of Lactate in the Brain

#### L-Lactate

The hypothesis of astrocyte-neuron lactate shuttle (ANLS) describing L-lactate shuttling between astrocytes and neurons is linked to glutamatergic signaling by Pellerin and Magistretti (40). A model opens the new insight for the L-lactate role in the brain and perfectly elaborates the mechanism of L-lactate how to serve as energy substrates. The ANLS switches on glutamate released by neuronal terminals and then taken up by astrocytes *via* the excitatory amino acid transporters 1 and 2 (EAAT1 and 2) to convert into glutamine or glutathione, which is activated by a gradient. This process stimulates the Na^+^-K^+^ pump to favor astrocytic mitochondrial acidification for launching glycolysis (21). Phosphofructokinase-2/fructose-2,6-bisphosphatase 3 (Pfkfb3) is a key positive modulator of glycolysis. Its expression and activity are high in astrocytes but not in neurons. These cell-specific expression and activity profiles result in the capacity of glycolysis being more active in astrocytes than neurons (26). The stored glycogen first decomposes into glucose-6-Phosphate (glucose-6P) catalyzed by glycogen phosphorylase. Then the glucose-6P gives rise to two molecules of pyruvate. Finally, pyruvate can be either converted to acetyl coenzyme A (acetyl-CoA) to be used directly by the astrocytic TCA cycle or transformed into L-lactate by lactate dehydrogenase 5 (LDH5) (26, 39).

Monocarboxylate transporters (MCTs) belong to the SLC16 gene family and facilitate the transmembrane H+-linked monocarboxylate, like lactate, pyruvate, and ketone bodies, to shuttle between astrocytes and neurons for the brain metabolic demands (26, 41). MCT1 is mainly located at the membrane of astrocytes, oligodendrocytes, and endothelial cells of blood vessels (42–44). The *Km* value of MCT1 for L-lactate approximates 3.5 mM. MCT4 is widely expressed at the membrane of astrocytes to cooperate with MCT1 in L-lactate efflux transport, in which the *Km* value for L-lactate is 34 mM. Compared with MCT1 and MCT4, MCT2 is specific in the membrane of neurons (45, 46) and has a higher affinity for L-lactate with the *Km* value of 0.7 mM (20, 47). The L-lactate from astrocytes or blood will be released into intercellular substances through MCT1 and/or MCT4, transported by MCT2 into neurons (30, 48), and then oxidized at the Krebs cycle (21).

**Figure 1:**
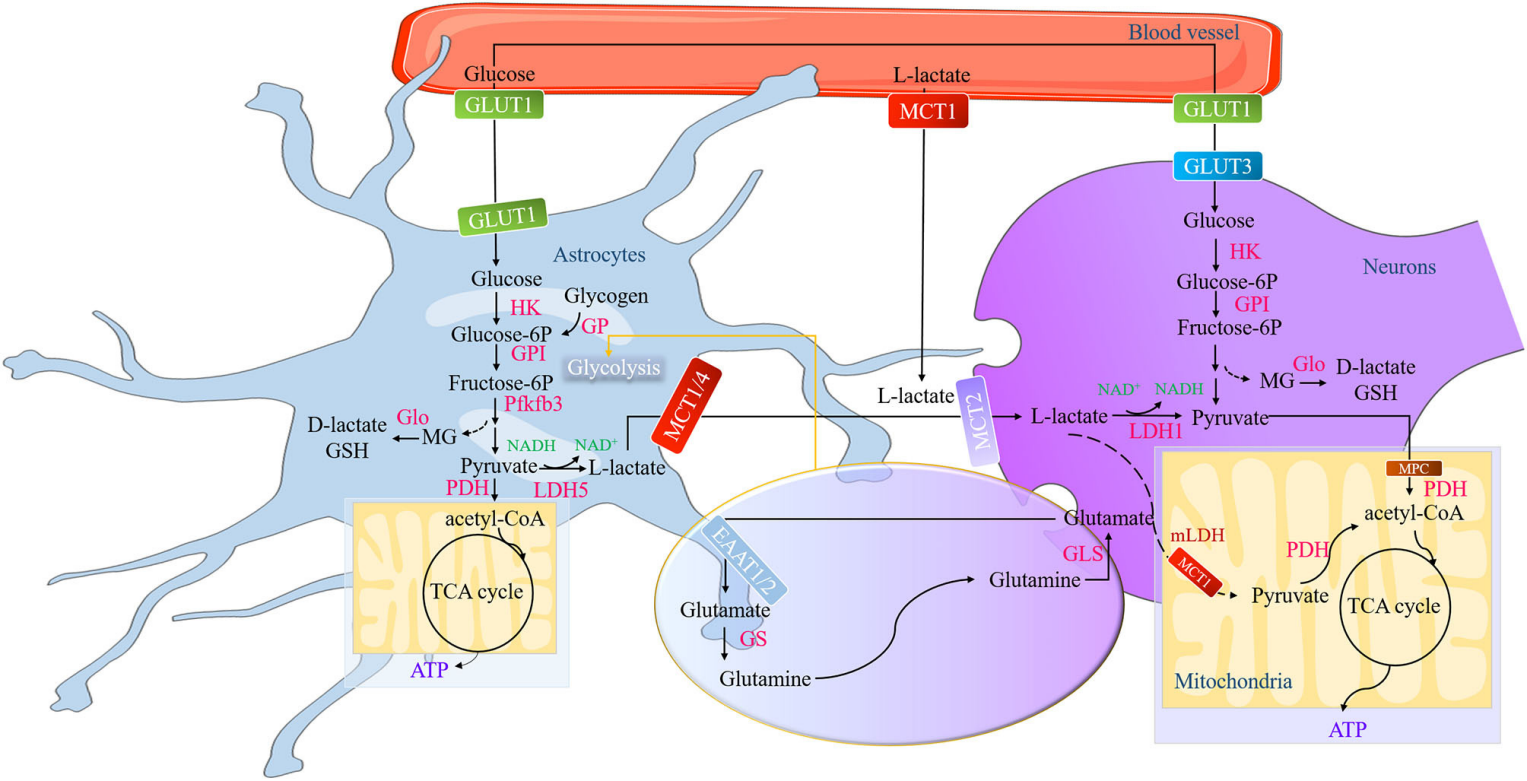
The formation and metabolism of lactate in the brain. The action of the glutamate-glutamine cycle switches on the glycolysis in the astrocytes, a process of glutamate released by neuronal terminals and then is taken up by astrocytes *via* the EAATs to convert into glutamine or GSH. Pfkfb3 (a key positive modulator of glycolysis) is a high expression in astrocytes and low in neurons, while the activity of PDH is low in astrocytes and low in neurons. These characteristics determine that astrocytes have strong glycolysis ability and neurons have strong aerobic oxidation ability. Astrocytic glycogen first decomposes into glucose-6P catalyzed by GP and finally into pyruvate through a series of catalytic reactions. During this process, a tiny amount of MG generates and then converts into endogenic D-lactate or GSH. The newly formed pyruvate can be either converted to acetyl-CoA for the astrocytic TCA cycle or transformed into L-lactate by LDH5. The L-lactate in the astrocytes and in the brain blood vessel is transported into the neurons through MCTs (MCT1, MCT2, and MCT4) and then oxidized at the Krebs cycle. EAATs, excitatory amino acid transporters; GSH, glutathione; Pfkfb3, phosphofructokinase-2/fructose-2,6-bisphosphatase 3; PDH, pyruvate dehydrogenase; Glucose-6P, glucose-6-Phosphate; GP, glycogen phosphorylase; MG, methylglyoxal; acetyl-CoA, acetyl coenzyme A; lactate dehydrogenase 5, LDH5; Monocarboxylic acid transporters, MCTs.

The L-lactate oxidation in neuronal cytoplasm into pyruvate by LDH1 is the first step to metabolic removal (1, 38). The newly generated pyruvate later crosses the mitochondrial inner membrane in favor of presumptive pyruvate transporters MPC (MPC1 and MPC2) and/or MCT1 (16, 49), to transform into acetyl-CoA for entering the tricarboxylic acid cycle (TCA) and therefore producing 14–17 ATPs per lactate molecule for maintaining neuronal activity (26). But the remarkable thing is that the MPC is only identified in yeast, drosophila, and humans (49). The molecular masses of MPC1 (15 kD) and MPC2 (14 kD) are akin to the findings in the experiment performed in rat liver and heart (50). That is to say, deficient evidence shows that the MPC exists in the neurons so far. The localization of MCT1 in mitochondrial inner membrane is not only verified in rat heart and muscle (51–53) but also in the rat cortical, hippocampal, and thalamic neurons (54). Besides deductive pyruvate transporters, L-lactate may be directly oxidized by mitochondria (in heart, muscle, liver, spermatozoa, and brain) (22, 55) in the wake of the concept “cytosol-mitochondria lactate shuttle” proposed by Brooks' team (53, 56–58). In this model, L-lactate enters into the mitochondrial intermembrane space through porins in the outer mitochondrial membrane. Then it will be oxidized by putative mitochondrial LDH (mLDH) (located at the inner mitochondrial membrane) into pyruvate (59). Several studies have confirmed that L-lactate can be taken up and metabolized by the mLDH in both astrocytes (human astrocytoma cells) (60) and neurons (rat cerebellar granule cells, cortex neurons, and hippocampal neurons) (54, 61). The evidence implies that cytosol-mitochondria lactate shuttle may occur in the brain (Figure 1).

#### D-Lactate

D-lactate exerts metabolically inert in mammals (62) because of the absence of specific cytosolic lactate dehydrogenase (D-LDH) to metabolize D-lactate into pyruvate (63, 64). This enzyme barely exists in mammals (24). Some evidence shows that MCTs can transport D-lactate to inhibit L-lactate out of astrocytes or into neurons despite the *Km* value of MCTs for D-lactate being far higher than L-lactate (65, 66). The rate of oxidation of D-lactate in the brain is considerably slower than that of L-lactate due to the rather low expression levels of D-LDH in the brain (36, 67). Since interfering with more efficient energy substrates (pyruvate and L-lactate) for mitochondrial use, the physiological function of D-lactate is now known as the competitive inhibitor of L-lactate (18). Moreover, D-lactate is confirmed to interfere with the pyruvate metabolism in the brain, which eventually impairs in state 3 and state 4 of mitochondrial respiration (63), its accumulation may have an adverse impact on energy metabolism and thus lead to toxicity (Figure 1).

## Lactate Intakes From the Food

Lactate, as the predominant end-product of lactose fermentation in the food (68), is produced by the lactic acid bacteria, namely, gram-positive and catalase-negative microorganisms. Such microorganisms are involved in the fermentation of a range of foods and beverages, such as dairy products, meat, fish, vegetable, sourdough, wine, and cider (68), which creates the specific flavors and aromas for the food and benefits human health (69). People can uptake lactate from cheese, yogurt, wine, fermented vegetables (69), and fermented oyster extract (70). Thereby, lactate will appear in the gut *via* the consumption of fermented foods. In addition, prebiotic, fiber-containing foods also are the way of the lactate intake, such as broccoli, brussels sprouts, cabbage, cauliflower, collard greens, kale, radish, and rutabaga (37). Since gut microbiota is likely to produce a racemic mixture, it is not surprising that L- and D-lactate are generated simultaneously following the above food intake (37). The disposal of lactate in the lower gut can be converted to acetate, butyrate, propionate, and succinate. Alternatively, the unverified idea of “gut-soma lactate shuttle” is another way for the disposal of lactate in the gut, in which the lactate production in the gut releases into the systemic circulation (37). The discovery of the sodium-dependent monocarboxylate transporter (SMCT) (including SMCT1 and SMCT2) is favorable evidence for supporting this hypothesis, which is located in the mouse digestive tract and involved in the transport of food-derived monocarboxylates, such as lactate (71). The other implicit clue is the phenomenon of the rise in the blood L-lactate after a carbohydrate diet (37, 72). Tappy's team finds that dietary fructose or the co-mixture (fructose and glucose) facilitate the L-lactate release into the systemic circulation (73, 74). Furthermore, glucose rooted in the oxidation of carbohydrate only provides 10–20% energy, while other carbohydrate energy sources like glycogen and L-lactate accounts for 70–80% in the condition of exercise (75). That is to say when blood glucose is supported by hepatic glycogenolysis and gluconeogenesis, L-lactate plays important role in carbohydrate energy substrate distribution (72). As blood L-lactate can be transported into the brain *via* the MCT1 (32, 33), net L-lactate uptake directly provides 12% of brain fuel (37, 75, 76). Besides, gluconeogenesis provides 45% of brain fuel. Thereby, L-lactate comprises 57% of the total brain energy source (75). Regarding D-lactate, it can be excreted in urine by renal (64) or feces by gut (37) and cannot be detected in the blood under normal physiological conditions (64). Its excessive accumulation can result in acidosis and irritation of the lower bowel. Furthermore, the release of D-lactate into circulation also cause neurotoxic effect (37). The clinical presentation of D-lactic acidosis is characterized by episodes of encephalopathy and metabolic acidosis (24).

## The Role of Lactate in Regulating Brain Functions

Lactate is reported to participate in the regulation of various brain functions in the terms of learning and memory (3), cerebral blood flow (4), neurogenesis (5, 6) and cerebral microangiogenesis (7), energy metabolism (8), neuronal activity (9–11), and neuroprotection (12–15). L-lactate and D-lactate are reported to exert similar or distinct effects on those functions.

### The Distinction Between L-Lactate and D-Lactate for Influencing Learning and Memory

#### The Effect of L-Lactate on Learning and Memory

**Figure 2:**
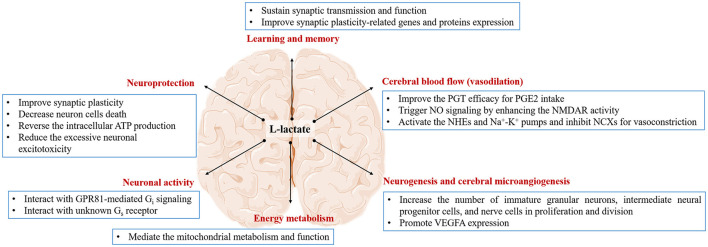
The effect of L-lactate and its possible mechanism on the brain. L-lactate is capable of regulating brain function in the terms of learning and memory, cerebral blood flow, neurogenesis and cerebral microangiogenesis, energy metabolism, neuronal activity, and neuroprotection *via* different pathways.

The glycogen is necessary for long-term potentiation maintenance in the mouse Shaffer collateral-CA1 synapse (77). Shima et al. find that 4-week exercise can increase glycogen reserve, along with the elevated transport rate of L-lactate into the neurons, to ameliorate memory dysfunction in diabetic rats (78). Extracellular L-lactate rapidly increases in the rat hippocampus during spontaneous alternation. Intrahippocampal 50 nM L-lactate can not only enhance the memory in this task but will rescue impaired memory in the condition of glycogenolysis inhibition (27). This evidence shows that the use of L-lactate metabolically coupled astrocytes and neurons depends on the character of high-energy demands for memory consolidation and storage (30). The provision of L-lactate by astrocytes is proved to be a more generally important element of learning and memory processing than memory consolidation (29). Harris et al. find that the L-lactate produced by glycolysis, is required for memory acquisition but not for established memory in mice at the age of 9 months (79), suggesting that the main function of L-lactate is regulating the process of learning (80). Recent studies reveal the fact that L-lactate transporters are necessary for L-lactate mediating the memory process, especially MCT2. Inhibition of MCT1 or MCT2 can impair the rat reconsolidation of cocaine memory (81) or the mouse long-term memory formation (82). L-lactate replenishment will reverse the memory impairment in MCT1 or MCT4 knockdown of rat hippocampus (77). By contrast, L-lactate replenishment or even glucose supplement will not rescue the impaired memory once blocking MCT2 activity, which transports L-lactate into neurons (27, 77). In these studies, L-lactate in neurons is seen as the pyruvate donor, which produces ATP for neuronal energy demand (see section “Serves as an energy substrate for neurons”). Therefore, the transfer of L-lactate from astrocytes into neurons to support neuronal functions is necessary for memory consolidation (77, 83). The mechanisms may involve sustaining synaptic transmission and function (84–88) and regulating synaptic plasticity-related genes and proteins expression (89, 90). In facilitating the synaptic transmission and function, Tang et al. find that injections of 2 mM L-lactate into the locus coeruleus can activate an unidentified G_s_ receptor to evoke the NEergic neuronal excitability for norepinephrine release (85). Schurr et al. first find that L-lactate can replace glucose as a sole energy substrate for sustaining the normal synaptic function in rat hippocampal slices for hours (87). Lucas et al. declare that the physiological concentration of L-lactate can be utilized by presynaptic terminals as the energy for meeting the demand of maintaining functional presynaptic release sites (84). Herrera-Lo'pez et al. find that 1–2 mM L-lactate can induce glutamatergic synapse potentiation to promote the memory formation process in rat hippocampal CA3 pyramidal cells (86). As for modulating the synaptic plasticity genes and proteins expression, Margineanu et al. find that 20 mM L-lactate treatment can promote various plastic plasticity and plastic activity-related genes in mouse cortical neurons through RNA sequence (89). Yang et al. find that L-lactate from 2.5 to 20 mM will potentiate the NMDAR activity to improve the immediate early genes (IEGs) expression in mouse cortical neurons, which benefits the form of learning and memory (90). Hayek et al. find that intraperitoneal injection physiological concentration of L-lactate (117 or 180 mg/Kg) for 1 month can promote the proteins and genes expression of brain-derived neurotrophic factor (BDNF) and IEGs to improve the mouse learning and memory ability (3) (Figure 2).

#### The Effect of D-Lactate on Learning and Memory

D-lactate is proved to impair the neonatal chick memory process in the way of intracranial injection (23, 91). The reasons ascribe inhibition of L-lactate uptake into neurons and interference with the astrocytic metabolism. Baker and Edwards find that bilateral administration of D-lactate in the lower dosage range of 1.75–2.25 mM inhibits memory retention after discrimination avoidance task from 40 min and onward, and the inhibitory effect sustains 140 min. And the scope of the validity period is from 10 min before to 20 min after the task (91). This time window is similar to the dosage of 10 nM (23). The observation indicates that the inhibitory effect of D-lactate on memory is valid for a certain period (23). Further to support this idea is the study of rodents. Michael et al. find that intracranial injection of a higher concentration of D-lactate (18 mM) at 30 min before the Y maze task seems to not affect memory retention (92). Scavuzzo et al. even find that subcutaneous injections of D-lactate (1 g/kg) impair memory at 15 min before the inhibitory avoidance (IA) task, whereas it significantly enhances the rat memory at 2 min after the training (80) (Table 1). Thereby, it is still debatable that the different concentration and time-point of D-lactate usage lead to the distinct results for memory up to date. The conflict results are puzzling what is the physiological role of D-lactate in learning and memory. In short, it will be interesting to figure out the mechanism of D-lactate on the memory at the level of molecules and physiology. More interestingly, the results of intracranial unilateral injection by Gibbs and Hertz also show that D-lactate injection into left intermediate medial mesopallium inhibits memory formation from 10 min before task, whereas injection into the right hemisphere works from 10 min after task (23), implying that the time window of D-lactate on inhibiting memory formation varies from the part of the brain hemisphere.

**Table 1:** The effect of D-lactate on the brain function.

**Brain function**	**Model**	**Method of administration**	**Concentration**	**Intervention time**	**Effect**
**Learning and memory**	Neonatal chick (91)	Tail intravenous injection	1.75–2.25 mM	The scope of validity period from 10 min before to 20 min after the discrimination avoidance task	Impair memory
	Neonatal chick (23)	Intracranial injection	10 nM	The scope of validity period from 10 min before to 20 min after the discrimination avoidance task Injection at 20 min before or 25 min after the task	Impair memory No adverse effect on memory
	9~10-week-old rats (92)	Intracranial injection	18 mM	Injection at 30 min before the Y maze task	No adverse effect on memory retention
	6~11-week-old rats (80)	Subcutaneous injection	1 g/kg	Injection within 2 min following IA task Injection at 15 min prior to IA task	Enhance memory Impair memory
**Neuronal activity**	Glutamatergic neurons and GABAergic neurons (9)		5 mM		Decrease the spontaneous calcium spiking frequency
**Neuroprotection**	tMCAO model Hippocampal slices in OGD model (12)	Intravenous injection	1 μmol/g 4 mM	Injection at 45 min after tMCAO 48 h after OGD	Decrease the infarct volume Reduce neuronal cell death
	NMDAR-induced neuronal excitotoxicity (93)		10 mM	22 min before adding glutamate.	No alteration in excitotoxicity
	Glutamate-induced neurotoxicity model (94)	Infusion	6 mM	Combination with containing glutamate perfusion medium	Aggravate the cortex lesion area

### L-Lactate Increases Cerebral Blood Flow (Vasodilation)

For the last few years, the function of L-lactate regulating cerebral blood flow (CBF) has been proposed in several studies (4, 95–98). First, a hypoxia-induced increase of L-lactate can improve the prostaglandin transporter (PGT) efficacy for vasodilator prostaglandin E2 (PGE2) intake in the brain (4). Second, exercise is reported to trigger the N-methyl D-aspartate (NMDA) receptor/nitric oxide (NO) signaling for realizing the hippocampal functional hyperemia during neural activation (99). Previous evidence indicates the effect of L-lactate on enhancing the NMDAR activity in cortex neurons (90). Moreover, when the porcine second-order retinal arterioles are pressurized to no flow, 10 mM L-lactate can induce the release of NO synthase to activate the downstream guanylyl cyclase/cGMP signaling, which opens the K_ATP_ channels for vasodilation in retinal arterioles (100). Contemplating all this, it is possible for L-lactate to dilate the brain microvessels for increasing CBF *via* NO biological effect. During hypoglycemia, the CBF increase can be considered neuroprotective since it is an attempt to increase capillary glucose concentration for improved glucose supply to the brain (101). Third, L-lactate has the autoregulatory vascular function in switching the pericyte response from contraction to dilation in the retinal vasculature. In the normal physiological state, L-lactate activates the Na^+^/H^+^ exchangers, inhibits Na^+^/Ca^2+^ exchangers, and excites Na^+^-K^+^ pumps to trigger pericyte vasoconstriction regardless of the L-lactate concentration (Figure 2). While under the hypoxic condition, 20 mM L-lactate induces mural cell relaxation throughout the retinal vasculature, from the arterioles to the capillaries (102). Exploring the L-lactate-induced vascular reaction when dealing with the physiological and pathological situation, may be crucial for aiding in our understanding of neurovascular coupling, enhancing brain functions, and even treating multiple brain diseases.

### L-Lactate Benefits the Process of Neurogenesis and Cerebral Microangiogenesis

Neurogenesis is important for memory and learning. Lambertus et al. find that the 7-week exercise or 18 mM L-lactate can induce neurogenesis in the mouse ventricular-subventricular zone but not in the hippocampus (5). Notably, the author mentioned that neurogenesis in this zone contributes to the olfactory memory, which may provide contextual clues to the spatial-visual memory controlled by the hippocampus (5). Controversially, Vachnish et al. find that 6-week 13–17 mM L-lactate can promote mice hippocampal neurogenesis (6). The distinct results are probably related to the selection of neurogenic markers. The former refers to DCX and Ki-67 to represent neurogenesis, which shows immature granular neurons and intermediate neural progenitor cells, respectively. The latter chooses BrdU as the neurogenic marker, which shows nerve cells in proliferation and division. The different markers are on behalf of different stages of neurogenesis (103). The above evidence suggests that the neurogenic effect of L-lactate is tissue-specific. For example, the effect of L-lactate on the ventricular-subventricular zone depends on the GPR81 action but the hippocampus depends on the MCT2 metabolic action. Since neurogenesis is highly correlated with cerebral microangiogenesis, there is no doubt that angiogenesis also may be influenced by L-lactate in the brain. The direct evidence is supported by Moland's team. The study declares that 7-week 18 mM L-lactate is capable of increasing the density of microvessels in the dentate gyrus of the hippocampus along with a higher VEGFA expression. Furthermore, this effect depends on the GPR81 action (7) (Figure 2). In the further study, figuring out the approaches of L-lactate how to deal with neurogenesis and cerebral microangiogenesis (metabolic pathway, lactormone action, or both), may be worthiness for understanding the physiological role of L-lactate in brain function regulation.

### L-Lactate Influences Brain Energy Metabolism

Mitochondria are best known for their role in the generation of ATP that supplies eukaryotes with energy to serve their cellular needs (104). Recent studies report that L-lactate can also mediate various mitochondria-related genes. A novel discovery is first reported by Brooks's team in L6 cells (105). They find that 20 mM L-lactate can upregulate 79 genes involved in mitochondrial metabolism (MFN1, MFN2, PGC-1α, NRF2, LDHb, ATP5g1, NADH-dh, SDH, and TIM) and oxidative stress (GPX1, Glrx2, Glrx5, Prdx2, and Txndc12) (105). The evidence is also verified in the brain. For example, intraperitoneal injection of 18 mM L-lactate for 14 consecutive days will promote the PRC mRNA expression and the mtDNA levels (8). 20 mM L-lactate pretreats SY5Y cells can reverse high-concentration hydrogen peroxide (H_2_O_2_)-induced oxidative stress injury, including NRF2 expression improvement and mitochondrial membrane potential potentiation (106). The biological effect of L-lactate mediating mitochondrial metabolism and function is also in the primary mouse neurons. A total of 15–20 mM L-lactate can improve mitochondrial fusion (OPA1, MFN1, and MFN2), inhibit mitochondrial fission (DRP1 and FIS1), and promote biogenesis (PGC-1α, NRF2, TFAM, and mtDNA) (3, 107) (Figure 2). Besides, “cytosol-mitochondria lactate shuttle” also elaborates the role of L-lactate as the substrate source of mitochondrial ATP production (53, 56–58). Thus, L-lactate ought to have a close relationship with mitochondria in the brain. Considering the fact that multiple brain diseases in a large part are also associated with the energy crisis, the application of L-lactate in an animal-related experiment to shed light on the effect and mitochondria mechanism on brain energy metabolism as early as possible should have a great significance of the novel treatment in clinic brain diseases.

### L-Lactate and D-Lactate Influence Neuronal Activity

The previous study demonstrates that lactate interacting with its receptor GPR81 operates negative feedback on neuronal activity. Application of 5 mM L- or D-lactate reversibly diminishes the calcium transient frequency by more than 50% in both principal and GABAergic neurons (Table 1). Moreover, the activation of GPR81 can also mimic a similar potency as the lactate (9). In fact, lactate can bind to GPR81 to couple with the G_iα_ subunit to inhibit the intracellular adenylate cyclase (AC)-cyclic adenosine monophosphate (cAMP) cascade signal that contributes to the reduction of exocytosis (10, 11). In addition, the GPR81 activation can bind to the G_iβ_ subunit to regulate the activity of phospholipase C (PLC) and thus inhibit neuronal excitability (10) by inducing a hyperpolarization for potassium (K^+^) indrawal or activating the GABA receptor for exocytosis reduction (9). Interestingly, 5 mM L-lactate and 0.56 mM 3,5-dihydroxybenzoic acid (3,5-DHBA) (one of the GPR81 agonists) reduce the firing frequency of CA1 pyramidal cells, whereas a higher level of L-lactate (30 mM) and 3,5-DHBA (3.1 mM) increase the firing frequency (11). In the locus coeruleus, 2 mM L-lactate has rather an excitatory effect on the NEergic neurons by activating an unidentified Gs receptor, which will be abolished by the D-lactate (85) (Figure 2). Given these, we speculate that the effect of L-lactate on neuronal activity may depend on the L-lactate concentration, the types of neurons, and the types of receptors. Knowledge of the physiological effects of different concentrations and lactate isomers on the activity of different neurons, application of lactate is a potential therapeutic way to improve the complex neurological symptoms of epilepsy, which disease is characterized by neuronal hyperexcitability and sudden, synchronized electrical discharges (47, 108). In fact, some studies have focused on the application of L-lactate in treating epilepsy (see section Epilepsy).

### The Neuroprotective Role of L-Lactate and D-Lactate

Lactate has been reported the role of neuroprotection in multiple brain disease models, however, the effect of the isomer of L- and D-lactate is split depending on the disease patterns. As known, transient middle cerebral artery occlusion (tMCAO) *in vivo* and oxygen and glucose deprivation (OGD) *in vitro* are the desired models for stroke. In the tMCAO model, L-lactate treatment can ameliorate neurological deficits after ischemia by inducing sustained neuroprotection for up to two weeks (14, 15). And as for the D-lactate treatment, it is also reported to exert neuroprotection by decreasing the infarct volume and ameliorating neurological deficits by working as the energy substrate or activating the receptor GPR81 (12). In the OGD model, the effect of ameliorating neuronal injury is possessed by both L-lactate and D-lactate (12–14). Hence, L- and D-lactate seem to exert a neuroprotective role in stroke.

In the human and rodent traumatic brain injury (TBI) model, evidence shows that endogenous produced brain L-lactate or exogenous L-lactate replenishment can facilitate neurologic recovery by improving synaptic plasticity (109) and regulating the brain metabolic state (76, 110–114) (see section “TBI”). However, the level of blood D-lactate is adopted as the biomarker of TBI degree. This is because that D-lactate is one of the microbe-dependent metabolites which will pass through the destroyed intestinal barrier into the blood when TBI causes gut dysbiosis (115, 116).

In addition, L-lactate but not D-lactate can reduce the excessive NMDAR-induced neuronal excitotoxicity, which is common in some acute brain pathologies. 10 mM L-lactate can improve the intracellular ATP production to stimulate metabotropic purinergic receptor P2Y for activating the PI3K pathway and opening the K_ATP_ channels, as a result of decreasing the intracellular Ca^2+^ concentration (93) (Figure 2). Furthermore, in the high concentration of glutamate-induced neurotoxicity model, infusion of 6 mM L-lactate can obviously reduce the rat cortex lesion from 6.05 ± 0.64 mm^3^ to 4.16 ± 0.43 mm^3^. On the contrary, the same concentration of D-lactate aggravates the lesion area from 2.7 ± 0.4 mm^3^ to 4.4 ± 0.5 mm^3^ (18) (Table 1). With a view to the metabolism of lactate and the universality of neuroprotective effects in the brain, L-lactate is a preferred research direction and practical value of the application and treatment in neuroprotection.

## Lactate Is the Potential Therapy for Multiple Brain Diseases

In the preceding decades, lactate was long considered as a waste product or even a sign of cerebral harm. As the proposal of the novel opinion such as “lactate shuttles” (37) and “lactormone” (22), these theories transform our stereotype and gradually unlock the mystery of lactate in physiological and pathological states. Recently, lactate (especially L-lactate) is postulated to protect the brain in several pathologic conditions, such as diabetic encephalopathy, Alzheimer's disease (AD), stroke, TBI, and epilepsy.

### Diabetic Encephalopathy

Diabetes mellitus often hurts brain health, which accompanies the symptoms of brain functional decline (117–119), especially cognitive impairment and vascular dementia (120). This symptom is called “diabetic encephalopathy” (121). Evidence shows that the brain lactate falls in response to hypoglycemia in diabetes (122, 123). Intravenous injection of L-lactate is capable of reducing the symptomatic and adrenaline responses to hypoglycemia and simultaneously alleviating hypoglycemia-induced cognitive dysfunction in diabetic patients (124–127). In addition, intracerebroventricular administration of 200 nM L-lactate can also mitigate the deterioration of memory consolidation *via* recovering the amplitude of sharp-wave ripples (SWRs) in diabetic mice (128). These results directly demonstrate that the L-lactate supplement benefits diabetic brain function. Another indirect evidence is that an increase of L-lactate transporter MCTs in the cortex and/or the hippocampus, especially MCT2 (transport L-lactate into neurons), is observed to be capable of ameliorating the cognitive impairment in diabetic encephalopathy (32, 78, 129). It is conceivable that L-lactate uptake enhancement supported by MCTs may improve diabetic encephalopathy. However, some studies show the phenomenon that the L-lactate increases in the brain regions of corpus callosum and hippocampus in diabetic encephalopathy (130–132). One possibility is the low utilization rate of L-lactate during diabetic encephalopathy (133, 134). The convincing evidence listed is that the L-lactate is secreted to increase in astrocytes but not in neurons in the condition of a high-glucose environment (134, 135), implying that the transport of L-lactate into neurons is hampered and thus the elevated brain L-lactate is observed in diabetic encephalopathy.

### AD

Alzheimer's disease (AD) is one of the most common forms of brain disease (136), characterized by a series of neuropathological changes, such as cognitive dysfunction. Cognitive impairment in AD is associated with recessionary energy metabolism (137). Whereas, L-lactate seems like an important energy substrate for memory formation in connection with energy supply (19, 27). In fact, decreased L-lactate content is observed in the cerebral cortex and hippocampus in AD rodent models (138–140). Nevertheless, elevated L-lactate levels occur in aging models (94, 141, 142) and AD patients (143). The increased lactate dehydrogenase A (LDH-A)/lactate dehydrogenase B (LDH-B) gene activity ratios (94) and downregulated MCTs (MCT1, MCT2, and MCT4) are due for the opposite results (139, 140). This is because that increased brain L-lactate production owing to the LDH-A/LDH-B ratio but blockage of L-lactate transport from glia to neurons, resulting in L-lactate deficit in neurons. Inferior L-lactate utilization rate leads to energy deficiency in neurons and thereby exacerbates the progression of neuronal injury, including cognitive impairment during the pathological process of AD or aging.

Regarding D-lactate, a study reports that D-LDH, located at the inner side of the rat liver mitochondrial inner membrane (MIM), oxidizes D-lactate to pyruvate in the mitochondrial matrix (144). This process will improve oxidative phosphorylation (OXPHOS) efficiency, synthesis, and efflux of biosynthetic precursors from mitochondria, balance the cytosolic and mitochondrial GSH pools, and enhance the NADPH production in the cytosol. All of these can contribute to mitochondrial energy production (145). Nevertheless, the D-LDH isoforms in both human and mouse brains show a rather weak signal (67). Based on this evidence, we speculate that moderate D-lactate combined with D-LDH supplement may target a novel mitochondrial energetic mechanism for AD treatment.

### Stroke

Stroke, one of the important factors causing neurological morbidity and mortality, is most characterized by ischemia and infarcts involving the territory of the middle cerebral artery (146). Berthet et al. adopt different concentrations of L-lactate treatment after OGD in the rat hippocampal slices. The results show that, immediately after OGD, the low dose treatment of L-lactate (4 mM) significantly inhibits the hippocampal neuron death, while L-lactate at the 20 mM dose aggravates the neuronal injury (14). Whereas, during OGD, only a high dose of L-lactate at 20 mM is the capacity of reversing the ATP decline to prevent cell death in primary cultured neurons and N2A cells, but not the low dose at 1 mM (13). Hence, the neuroprotective effect of L-lactate on stroke may be related to the time points for intervention or the types of neuron cells. Further study shows that L-lactate can reduce the size of the lesion area to promote neurological rehabilitation in the way of intracerebroventricular injection of 100 mM L-lactate (14) or tail vein injection of 200 mM L-lactate (15) immediately after tMCAO. They speculate that the ischemia-induced lactate increase may initially have a neuroprotective effect on the brain, but also may cause lactic acidosis when the lactate concentration reaches higher levels (14). Therefore, different concentrations of L-lactate may have different regulatory effects on brain metabolism and function under different physiological metabolic states. The complex metabolic characteristics of L-lactate bring immense challenges for revealing its role in brain function (14). Soon afterward, their team finds that similar neuroprotection occurs in D-lactate. The results suggest that intravenous injection of 1 μmol/g D-lactate will decrease about half of the infarct volume in tMCAO of the mice. Moreover, in the OGD model, 4 mM D-lactate treatment can also reduce about 50% neuronal cell death in the rat hippocampal slices (12). Future studies need further excellently clarify the mechanism of how lactate performs neuroprotection although it has fully considered the dual role of lactate as the energy substrate (pyruvate replacement) or signal molecular (receptor agonist replacement). So far, two results are responsible for explaining the neuroprotective effect of lactate on stroke. First, the accumulated lactate is transported by MCTs into the neurons and utilized as the alternative energy substrate immediately postischemia (147) in favor of sustaining neuronal integrity (148) and delaying the neuronal damage (149). Second, lactate may interact with the known receptor-GPR81 or unknown putative G_s_-type receptors to resist ischemic injury. It is worth noting that the evidence and characterization of GPR81 are still disputed in stroke. For example, Castillo et al. find the 3,5-DHBA treatment, one of the GPR81 agonists, can reduce neuronal cell death in OGD (12). In contrast with the study, Shen et al. find that inhibition of the GPR81 activity can promote the ERK1/2 signal to copy with apoptosis in neurons (13). Moreover, a newly published study also reports that the GPR81 agonists (3Cl-5OH-BA and 3,5-DHBA) do exert no neuroprotective effect on stroke mice whether intravenous administration or intracerebroventricular administration (150). Applying the genetic tools for GPR81 knockout may be a preferred protocol to definite the role and the mechanism of this receptor in the stroke.

### TBI

Traumatic brain injury (TBI) is a type of brain injury acquired from an external force that inflicts devastating effects on the brain vasculature and neurons (116). It is always reported to face an energy crisis in the brain in the fact of glucose uptake suppression following cerebral injury. Although glucose is the preferred fuel of the brain, it may result in odious insults, such as hyperglycemia and infection or mortality events increment (151–153). As in previous studies, many TBI patients have observed the increased net L-lactate uptake after-hours postinjury (76, 154) without hyperglycemia (110), implying the protective function of L-lactate in TBI. Increasing blood arterial L-lactate to supraphysiologic range (4–5 mmol/l) in the early phase of brain injury can accelerate the recovery of neural function through cerebral perfusion and brain glucose availability improvement (155). Brooks and Martin find that endogenous L-lactate, induced by TBI, can support brain metabolism indirectly or directly (76). On the one hand, endogenous lactate is reported to be related to accelerating hepatic and renal gluconeogenesis to make glucose available for essential organs like the brain. On the other hand, elevated blood L-lactate may provide substrate directly to the injured brain in link with the L-lactate shuttle (114). Through isotope tracer, ~70% carbohydrate (direct lactate uptake and indirect glucose uptake from lactate) is provided for the TBI brain (113), suggesting the importance of endogenous L-lactate generation as the energy supply in TBI. In addition, exogenous intraperitoneal injection of 500 mg/kg L-lactate before 30 min of TBI is reported to alleviate the brain injury-induced neurological deficits by promoting the neuronal plasticity proteins (PSD95, GAP43, and BDNF) (109). Intravenous infusion of 5 mM L-lactate is also verified to utilize with sparing of cerebral glucose along with a reduction of brain glutamate and intracranial pressure after TBI, which implies the benefit of cerebral metabolic and hemodynamic effects (110). A total of 100 mM L-lactate infusion is shown reduced lesion volume at day 2 after cerebral cortical impact through slightly regulating cerebral blood flow (111). Hence, L-lactate, as the alternative source of glucose, has dream prospects to improve the outcome of posttraumatic brain damage.

Recently, the lactate receptor GPR81 is also reported to potentially participate in the pathological process of TBI. The evidence is that the increased GPR81 gene expression lasts at least 28 days after TBI injury in lesion areas of the cerebral cortex and hippocampus (156). Coincidentally, L-lactate further enhances the expression of GPR81 in the ipsilateral cortex and hippocampus 24 h after TBI (109). These results show that L-lactate may not only be the brain fuel, but also the possible upstream molecule that interacts with the receptor to activate or inhibit the downstream molecular signals for neuroprotection in the TBI.

### Epilepsy

Epilepsy is a common neurological emergency with considerable associated healthcare costs, morbidity, and mortality, which is often causing neuronal and glial damage (157). During epilepsy, L-lactate is observed to rise in the brain regions of cerebral gray and white matter (158), cerebrospinal fluid (159), cerebral cortex (160), and hippocampus (161). Previously, one research has proved that the elevated L-lactate level in tissues can persist for approximately 1 h (161). In some cases, the L-lactate level can even reach 6 mM (162). Jorwal and Sikdar find that 6 mM L-lactate application reduces the spike frequency and hyperpolarizes the subicular neurons in rat hippocampal slices, suggesting an anticonvulsant effect of L-lactate. Further electrophysiological recordings reveal that L-lactate induces the G_iβγ_ subunit activity to expedite the inwardly rectifying potassium (GIRK) channel. They also point out an interesting phenomenon that normal brain concentration of L-lactate (2 mM) and even up to 6 mM have few effects on rat hippocampus neural activity in the absence of epileptiform activity. This fact reminds us that the important role of elevated brain L-lactate in epilepsy (163). It is worth thinking that since there is no negative effect of excessive brain L-lactate on the mean spike frequency and membrane potential of subicular pyramidal neurons in the normal physical condition, what physiological effects can this L-lactate have after being metabolized or working as the neurotransmitter? Considering the fact that and concentration of 6 mM L-lactate is sufficient to activate its receptor GPR81, which is also known to couple to G_iβγ_ subunit to inhibit the neuronal activity (10). Another function of GPR81 is to couple to the G_iα_ subunit to inhibit the AC-cAMP cascade signal, as a result, the decrease of neuronal excitement (11). However, unlike L-lactate, 3,5-DHBA does not result in hyperpolarizing subicular neurons (10). Therefore, it is likely that L-lactate may interact with other unknown G_i_-type proteins. L-lactate-receptor signals may be the novel target for antiepileptic treatment.

## The Neurobiological Role of L-Lactate in the Brain

### Serves as an Energy Substrate for Neurons

**Figure 3:**
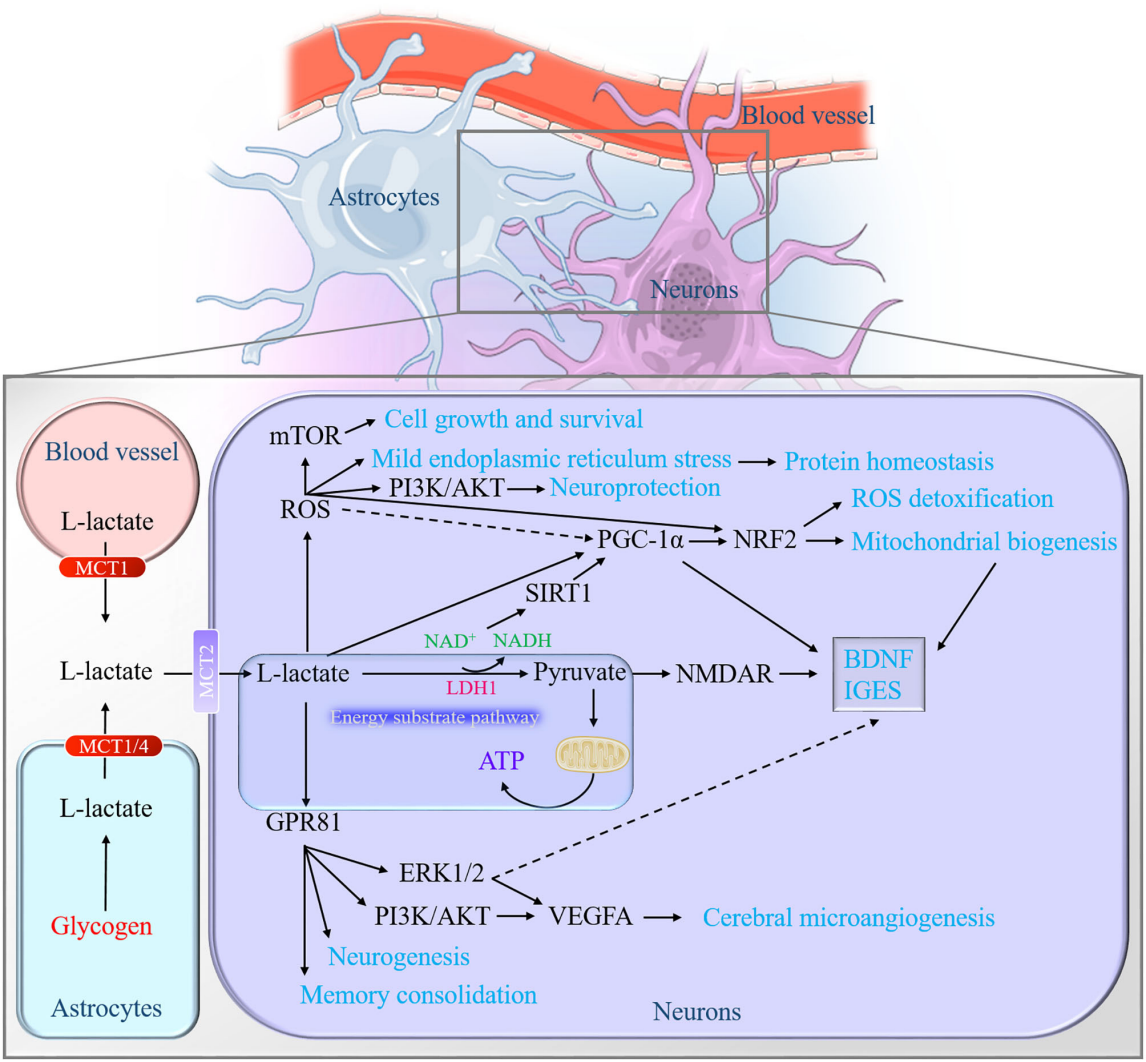
Graphical summary of the neurobiological role of L-lactate in the brain. On the one hand, L-lactate serves as an energy pool for neurons to facilitate mitochondrial energy production through converting into pyruvate. On the other hand, L-lactate may act as the role like the hormone, defined as lactormone. It triggers multiple downstream signaling cascades *via* influencing the intracellular NADH/NAD^+^ ratio, mediating the ROS generation, and activating the receptor GPR81. NADH, nicotinamide adenine dinucleotide; ROS, reactive oxygen species; GPR81, G_i_-protein-coupled receptor 81.

Astrocytic derived-L-lactate has been proposed to serve as an energy pool for neurons (40). In the presence or absence of oxygen, glycogen in the astrocytes can be broken down into L-lactate (30). An interesting phenomenon that manifests the role of L-lactate in neuronal energy metabolism is that the levels of glycogen in various brain regions (cortices, hippocampus, brainstem, epencephalon, and hypothalamus) are constant during the first hour of moderate-intensity exercise, while the levels of glycogen will decrease approximately 50% in the several hours that follow. Accompanied with the decreased glycogen levels are the elevated L-lactate levels in these regions (164). A similar phenomenon exists in the exhaustive swimming model, especially in the hippocampus (165). L-lactate oxidation metabolism is preferentially located in neurons by using the 3-[^13^C] lactate label (166). Furthermore, when increasing the plasma L-lactate levels, the cerebrum tends to reduce glucose utilization and prefers to utilize L-lactate by using the 1-[^11^C]L-lactate and [^18^F]fluorodeoxyglucose (FDG) for local cerebral metabolism measurement (148). The formed L-lactate in the brain is a crucial aerobic energy substrate that enables neurons to endure activation (167). Another evidence for L-lactate is the energy substrate of neurons is that the capacity of sustaining the presynaptic transmission function in the lack of glucose. The interesting note is that the transport of L-lactate into neurons is significant for ATP production to satisfy the energy demand under physiological or hypoxic conditions (28, 84, 168) (Figure 3).

### Signal Pathways Regulation as a Lactormone

In addition to being an energy substrate, L-lactate is also suggested its role as a signal regulator molecule or neurotransmitter since the proposal of “lactormone” (22) and the confirmation of its endogenous receptor GPR81 in mice brain (169).

#### Influences the Intracellular NADH/NAD^+^ Ratio

The catalytic action of LDH1 in neurons allows L-lactate to convert into pyruvate. This process of reducing NAD^+^ to NADH is supposed to influence NMDAR excitability (16, 93, 170). It is L-lactate, but not pyruvate, that generates NADH to enhance the NMDAR activity, which increases the intracellular Ca^2+^ and then promotes the expression of plasticity-associated genes (90). Along with the changes of redox state (NADH/NAD^+^ ratio), L-lactate is permissible to activate the NAD^+^-dependent histone deacetylase Sirtuin1 (SIRT1) (3, 171). SIRT1 deacetylates and amplifies the activity of the peroxisome proliferator-activated receptor-gamma coactivator-1 alpha (PGC-1α), which is known to mediate the BDNF expression (3) and mitochondrial biogenesis (172, 173). For example, Hayek et al. find that L-lactate can induce the expression of SITRT1 to activate the PGC1-1α, which will contribute to BDNF signals and some immediate early gene (IEGs) as a result in the mouse neurons (3). Our experiment also finds that a high concentration of L-lactate promotes the PGC-1-NRF2 signal axis and subsequently boosts mitochondrial biogenesis in mouse primary hippocampal cells (107) (Figure 3). However, Lezi et al. find that 14 consecutive-day-intraperitoneal injections of 18 mM L-lactate do not affect the PCG-1α-mediated mitochondrial biogenesis in the mouse brain (8). This may relate to the short administration time or the examination of the whole brain.

#### Activates Reactive Oxygen Species-Related Signals

It is well-known that excessive reactive oxygen species (ROS) production is considered a cause of several pathological conditions, which are harmful to the cells. Yet, low ROS production may be relevant to cellular activity under physiological conditions (174). Moreover, the moderate elevation of ROS can even protect against oxidative damage through the induction of antioxidant and detoxification enzymes (175).

Hashimoto et al. first report that L-lactate is able to stimulate some of the ROS-sensitive transcription factors' activity in L6 cells. Their research also reports that the PGC1α, known as a master coordinator of mitochondrial biogenesis, is activated in a high concentration of L-lactate treatment which is believed to be regulated by ROS and H_2_O_2_. Then the activated PGC1α interacts with transcription factors for mitochondrial gene expression, including cAMP-response element-binding protein (CREB) and nuclear respiratory factor (NRF)-2 in L6 cells (105). L-lactate metabolism also potentiates the phosphorylation of adenosine 5'-monophosphate (AMP)-activated protein kinase (AMPK) to activate PCG-1α signals for mitochondrial biogenesis and inhibits the phosphorylation of mTOR for mitophagy *via* mild production of ROS in skin fibroblasts (175). Recently, a similar phenomenon occurs in neurons. In SY5Y cells, 20 mM L-lactate is proved to induce moderate ROS for antioxidant stress and cellular homeostasis improvement, for instance, activating mTOR signaling for cell growth and survival, phosphatidylinositol 3-kinase (PI3-K)/protein kinase B (AKT) signaling for neuroprotection, mild endoplasmic reticulum stress (ERS) for protein homeostasis, and NRF2 signaling for ROS detoxification (Figure 3). Interestingly, it is an extremely high concentration of L-lactate (100 mM) but not lower or physiological concentration of L-lactate (50 and 10 mM) exerts antiaging phenotypes when *Caenorhabditis elegans* suffer from oxidative stress. Furthermore, 10 mM L-lactate suffice to resist stress and increase their lifespan (106).

Noteworthy, it is not known how L-lactate is ready-witted enough to mediate and maintain moderate ROS production for safeguarding cells when a high concentration of H_2_O_2_ provokes excessive ROS. One of the reasons may be associated with the potential ability of L-lactate sensing and decreasing the mitochondrial H_2_O_2_ production accurately to ensure moderate ROS production (175). Another hypothesis of L-lactate mitochondrial metabolism may explain the ROS signals activation. Bari et al. find that L-lactation can generate the H_2_O_2_ production *via* the inner membrane of putative L-lactate oxidase (LOX) in the pure rat liver mitochondria (176). Hence, L-lactate metabolism in mitochondria is likely to produce partial ROS and then activate its related signals if there exists analogous oxidase located at the mitochondrial inner membrane in the neurons. The evidence supports this hypothesis is that a mitochondrial lactate oxidation complex (mLOC) (including MCT1 or MCT2, LDH, and COX) is present in the neurons discovered by Hashimoto et al. (54). However, how L-lactate metabolism in neurons produces and regulates ROS in the subsequent process remains to be further investigated.

#### Interacts With the Receptor GPR81

GPR81 is a type of G protein (G_i_) coupled receptor and involves the metabolic process of some tissues and cells (177–179). So far, recent research has revealed that GPR81 regulates multiple signal pathways in tissues and cells, such as the extracellular regulated protein kinases (ERK1/2) (7, 180–182), nod-like receptor family pyrin domain-containing 3 (NLRP3)/ nuclear factor kappa-B (NF-κB) inflammation (183, 184), peroxisome proliferator-activated receptor γ (PPARγ) (185, 186) and Wnt signaling pathway (187, 188).

In the brain, it expresses in the regions of the pituitary (177), hippocampus, cerebellum, and brain stem (169). Cell localization indicates that it widely distributes at neuronal synaptic membranes (169), implying that GPR81 may have an important role in regulating brain function. It is known as the endogenic lactate receptor in 2008 (189, 190). The efficiency of lactate on GPR81 has a wide range from 0.2 to 30 mM (191). Furthermore, partial activation of GPR81 by lactate requires only 0.2–1 mM (192). Therefore, lactate absolutely has the ability to activate the GPR81 in the physiological state (human blood lactate concentration range is 0.5–2 mM) (193). Needless to say, in the conditions of intensive exercise and hypoxia, the blood lactate level can even reach 20–30 mM (194, 195). In neuronal activity, the activation of GPR81 inhibits the excitability of neurons (9, 10). In cellular energy, the astrocytic glycolysis rates accelerate ATP production to meet the energy requirements of neurons when the GPR81 agonists stimulate the cells (196). In terms of cerebral microangiogenesis and neurogenesis, L-lactate and exercise enhance the PI3K/AKT and ERK1/2 signaling pathway to promote VEGFA protein expression and vascular density in the dentate gyrus of mice *via* GPR81 (7). In addition, the neurogenesis effect of both L-lactate and exercise on the ventriculosubventricular region is dependent on the GPR81 in the mice (5). As for the learning and memory, intraperitoneal injection of GPR81 agonist 3,5-DHBA before the inhibitory avoidance task will impair the memory of rats, while the injection after the training contributes to memory consolidation. The distinct effects may be attributed to the reasons that the activation of GPR81 in the forebrain after training, can promote slow-wave activity (SWA) and enhance the consolidation of previous experience, but it also impairs the ongoing learning process at the same time (80). The results suggest that GPR81 activation at different stages of training may have different functions of influencing learning and memory. Synaptic plasticity, induced by appropriate synapses, is both necessary and sufficient for the information storage of memory (197). Previous studies revealed that L-lactate could promote plastic plasticity-related proteins and genes expression *via* enhancing the NMDAR-mediated phosphorylation of ERK1/2 (89, 90). The interesting thing is the fact that GPR81 is upstream of the ERK1/2 (7) (Figure 3). Hence, GPR81 is likely to act as a sensor for L-lactate metabolism in neurons and participate in L-lactate-regulated synaptic plasticity (198).

## Conclusion

To summarize the current evidence, this review shows that lactate participates in coordinating various brain functions in both the healthy and diseased states. The involving mechanisms are far more complex than originally thought and further knowledge of the lactate operating, as outlined in this article, is a drop in the bucket to our understanding of brain physiological and pathological mysteries. L-lactate not only serves as the energy substrate but also acts as lactormone in the regulation of downstream cascaded signaling pathways. Considering the consensus that lactate is a common metabolite and readily available (food intake or exercise), knowledge of lactate's role in the brain may provide precise tactics for brain diseases involving lactate metabolism. It also may set new insight and ideas for studying the relationship between lactate metabolism and brain function improvement.

## Author Contributions

MC and HW drafted the manuscript. HS, RY, and LW assisted with drafting the tables and figures. XX suggested valuable advice for the article. WS and JH conceptualized the article and revised the final version. All authors read and approved the final manuscript.

## Funding

This work was sponsored by Shanghai Sailing Program (22YF1441600), the grant of the funding of Youth Fund Project of Research Planning Foundation on Humanities and Social Sciences of the Ministry of Education (20YJCZH001), China Postdoctoral Science Foundation (2021M702128), and the Scientific Research Foundation of SUMHS (SSF-21-03-008).

## Conflict of Interest

The authors declare that the research was conducted in the absence of any commercial or financial relationships that could be construed as a potential conflict of interest.

## Publisher's Note

All claims expressed in this article are solely those of the authors and do not necessarily represent those of their affiliated organizations, or those of the publisher, the editors and the reviewers. Any product that may be evaluated in this article, or claim that may be made by its manufacturer, is not guaranteed or endorsed by the publisher.
